# A mobile learning framework for higher education in resource constrained environments

**DOI:** 10.1007/s10639-022-11094-5

**Published:** 2022-05-24

**Authors:** Safiya Okai-Ugbaje, Kathie Ardzejewska, Ahmed Imran

**Affiliations:** 1 School of Information Technology and Systems, University of Canberra, Bruce, Australia; 2 Learning and Teaching Office, The University of Notre Dame, Sydney, Australia

**Keywords:** Mobile learning, Higher education, Technology-enhanced learning, Low-income countries (LMICs)

## Abstract

It is well documented that learning oppourtunities afforded by mobile technology (m-learning) holds great potential to enhance technology-enhanced learning in countries and communities with low socio-economic conditions where web-based e-learning has failed because of limited infrastructure and resources. Despite the potential for m-learning, its actual uptake has been low. The extant literature in this sphere provides some theoretical insight, with evidence of limited on-the-ground practical studies that often do not progress beyond the pilot phase. Failure to embed sustainable learning opportunities has been attributed to the absence of a contextual framework suitable for the heterogeneous nature of many developing countries. This paper thus presents an m-learning framework that considers the sociocultural and socio-economic contexts of low-income economies. The framework is based on a range of studies conducted over four years, including the outcome of two empirical studies conducted in a Nigerian university. Documenting the research underpinning the design provides practitioners and policymakers with a framework for a potentially sustainable strategy for long-term mainstream m-learning integration in higher education in low-income countries.

## Introduction

It is well documented that the required infrastructure and cost associated with establishing and implementing technology-enhanced learning (TEL), particularly e-learning - that is, education delivery and/or learning over networked computing devices has impacted some higher education institution’s ability to provide such learning opportunities (Eltahir, 2019; Hadullo et al., 2018; Kigotho, 2018; Olutola & Olatoye, 2015). This is especially so in countries classified as low and lower-middle-income economies (LMICs) (World Bank Group, 2018). At a more fundamental level, some of these countries’ educational systems are challenged by inadequate funding, leading to crowded classrooms and limited facilities and resources for effective teaching and learning (Ewiss, 2020; Kuchah, 2018; Suresh & Kumaravelu, 2017). It has also been widely recognised that despite these circumstances, the availability of mobile technology platforms in all countries regardless of economic status provides an opportunity for improved educational systems and TEL opportunities in the form of mobile learning (m-learning) (Briggs, 2014; Lamptey, 2020; Traxler & Vosloo, 2014). Nevertheless, m-learning, a form of TEL using portable handheld mobile devices (e.g., phones and tablets) to facilitate and enrich learning regardless of circumstance, time, place and context, has not been widely adopted (Bikanga Ada, 2018; Okai-Ugbaje et al., 2017). Barriers to adoption reportedly include the absence of policies to drive implementation; commitment by institutional leadership and educators’ attitude; resources, knowledge and skill, including the pedagogical knowledge to support m-learning; and contextual theories (Farley et al., 2015; Lamptey & Boateng, 2017).

The need for the adoption of m-learning, which appears to be a more practical solution to the realities of LMICs due to the deep penetration of mobile technology in the region and the prospects of mobile devices to enhance and enrich learning cannot be overemphasised (Lamptey, 2020; Mohammadi et al., 2020). The coronavirus (COVID-19) pandemic has buttressed this need as the suspension of face-to-face teaching and learning to curtail the spread of the virus led to increased uptake of synchronous and asynchronous online teaching and learning globally (Dhawan, 2020; Li & Lalani, 2020; Pokhrel & Chhetri, 2021). In many LMICs, despite pockets of remote learning opportunities, including broadcast education via television and radio (Vegas, 2020; World Bank Group, 2020a), the physical shutdown of institutions halted the majority of students’ education (Olisah, 2020; Thomas, 2020). For many, their education only resumed when they were able to return to face-to-face classes. The gap in education was a result of poor infrastructure, inadequate funding and a lack of requisite skills to conduct e-learning (Lawal, 2020; Zalat et al., 2021). The troubling result is that the COVID-19 pandemic further weakened an already struggling educational system (World Bank Group, 2020b), the long-term impact of which is yet to be determined.

Against this backdrop, this paper attempts to provide a pathway for the seamless integration of m-learning into existing educational practices within the contexts of LMICs, to enhance and enrich teaching and learning in higher education. Such opportunities may provide a reasonable compromise or substitute to continuing the delivery of education in the event of circumstances warranting the shutdown of face-to-face delivery. The pathway presented is an m-learning framework that aligns theory with practice in using mobile devices to facilitate teaching and learning. The framework considers the pedagogical, socioeconomic, sociotechnical and sociocultural contexts of LMICs. These considerations are important because successful technology adoption not only relies on the availability of the required infrastructure but most importantly, a contextual understanding of the ground realities to ensure technology interventions are fruitful (Imran et al., 2017).

The research presented herewith brings together the findings of a variety of studies conducted over a four-year period, culminating in the creation of the framework. This paper concentrates on demonstrating the shift from the current realities facing higher education in LMICs to a potentially sustainable technology-enhanced and student-centred learning practice. Following this introduction, the paper presents a review to demonstrate the gap in literature warranting the creation of a context-specific m-learning framework for LMICs. Then, the conceptual framework that provided the theoretical basis for the proposed framework leading to the resulting framework is explained. The subsequent sections present the study’s methodology, reflections on the findings and further research directions for researchers wishing to extend this work.

## Literature review

This section is comprised of three parts. The first provides an overview of m-learning adoption and implementation. This is followed by a critical review and analysis of existing m-learning models and frameworks focused on pedagogies and the learning environment. Finally, a conceptual framework informed by the critical review findings is presented.

### M-learning adoption and implementation

To encourage m-learning adoption and implementation, prior studies have attempted to define its attributes in the form of theoretical models and frameworks, as these denote analytical principles, concepts and ideas that explain phenomena, events or behaviour with a structure, outline or plan (Nilsen, 2015). Hsu and Ching (2015) reviewed 17 m-learning models and frameworks to find the pattern in m-learning research. The models and frameworks were grouped into categories based on the emphasis of each study. The findings showed a growing interest in the pedagogical aspect of m-learning and advocation for m-learning adoption. The review also revealed that most models and frameworks are derived from the context of educationally advanced countries. More recent studies have continued to show these trends. For example, Romero-Rodríguez et al. (2020), in a review of 19 m-learning studies from the context of higher education, most of which were published between 2019 and 2020, also found an increasing trend in the pedagogical aspects of m-learning. Further, the majority of studies were theoretical, as only four of the 19 studies were based on practical concepts. A narrower examination of m-learning studies from the contexts of LMICs showed that m-learning research is gaining momentum in the region (Kaliisa & Picard, 2017; Lamptey & Boateng, 2017). However, again, the vast majority of studies are abstract rather than practical and not underpinned by theory despite the importance of theory to guide educational interventions (Okai-Ugbaje et al., 2017). Lamptey and Boateng (2017) attribute the poor theoretical underpinnings to the absence of theories that consider low-income countries’ pedagogical and socio-economic contexts.

### Review and analysis of m-learning models and frameworks

This section presents a critical review and analysis of m-learning theories, focusing on m-learning models and frameworks on pedagogies and the learning environment. In addition to targeting studies focused on m-learning pedagogies, only those with the tangible outcome of a model or framework were considered. For an in-depth analysis, the underpinning theoretical and/or pedagogical approaches of each model or framework, target audience, relationship with other learning approaches (traditional face-to-face, d-learning, and e-learning) and whether the models/frameworks were evaluated or validated were noted. A summary of the findings is presented in Table 1.

**Table 1:** Analysis of relevant mobile learning models and frameworks

Authors	summary of model/framework	Underpinned by theory?	Target audience	Link with other learning approaches	Context/ location	Evaluated/ validated?	Key contribution	Takeaways from other studies for this research
Abu-Al-Aish et al. (2013)	Outlined a roadmap for pre-and post-deployment stages of m-learning	No	Higher education	e-learning	UK	Yes	Identified ongoing management and technical support, and continuous innovation to accommodate changes in m-learning as key factors for m-learning deployment	Broad stakeholder involvement
Ng and Nicholas (2013)	Took a holistic view on the requirements for sustainable ICT in educational institutions, emphasising that pedagogical sustainability is a key requirement for m-learning.	No	Secondary school	Not specified	Australia	Yes	Emphasised the involvement of all stakeholders including management, technical support team, and the wider community if m-learning is to be successful.	
Barker et al. (2005)	Proposed a model for developing countries based on reports from m-learning projects and recommendations from developed countries	No	Any learning institution (school, community centre)	Traditional learning environment	South Africa	No	Found collaboration and motivation as critical success factors for m-learning	Motivation: present content in ways that attract learners to the learning process
Irugalbandara and Fernando (2019)	A context aware adaptive framework for bottom of pyramid people to achieve sustainable livelihood through vocational education using mobile devices	Keller’s ARCS model of motivation theory	Adult learners (vocational education and training)	Not specified	Sri Lanka	Yes	Developed a strategy for personalised content delivery to increase motivation of local traders to acquire knowledge	
Bikanga Ada (2018)	Developed an m-learning pedagogical framework for assessment feedback to evaluate m-learning outcomes.	No	Higher education	Not specified	UK	Yes	Provided a platform for communication, dialogue, and feedback between students and educators applicable in any learning context and discipline	M-learning platform/design focusing on: Communication, dialogue, and feedback Authentic, personalised, and social learning experience Sociocultural considerations, adapt learning to local context
Kearney et al. (2012)	A pedagogical framework based on personalisation, authenticity and collaborative features of the mobile device in teaching and learning, with special consideration to time and space constraints.	Sociocultural theory, motivational theory, and constructivism	Teachers engaged in m-learning	e-learning	UK and Australia	Yes	Highlighted the unique attributes of the mobile device leading to authentic and personalised learning experience for learners, and the ability to connect and collaborate with other people and resources for effective learning	
Koole (2009)	Described m-learning as a process derived from the convergence of mobile technologies, human learning capacities, and social interaction	Activity theory and constructivism	Instructional designers and instructors Developers of mobile devices	Not specified	Canada	No	Considered the technocentric, pedagogical and interactive aspects of the mobile device for learning. Provided a checklist to guide the development and assessment of m-learning environments and aid in the development of future devices.	
Park (2011)	Modified the transactional distance theory to develop a new framework that elaborated on the characteristics of m-learning in the context of distance education.	Transactional distance theory, activity theory, and constructivism	Instructional designers and instructors	d-learning	USA	No	Explored the attributes of mobile technologies that support the individualised and social aspects of learning specifically suited for distance education.	
Peng et al. (2009)	A framework for ubiquitous knowledge construction to aid educators and researchers in examining the implications of m-learning	Constructivism and lifelong learning theories	Educators and researchers	e-learning	Taiwan	No	Re-defined m-learning in the light of ubiquitousness learning and knowledge construction	
Xue (2020)	A conceptual model for integrating mobile and emerging technologies into task-based language learning	The conversational framework	Second language instructors and learners	Not specified	Hong Kong	No	Produced a conceptual framework to enhance language teaching and learning	

The literature review findings are consistent with claims that there are limited m-learning models/frameworks from and in the context of developing countries (Hsu & Ching, 2015; Lamptey & Boateng, 2017; Romero-Rodríguez et al., 2020). While the overall number of m-learning models/frameworks is relatively low, the analysis has also shown that a comprehensive framework that is grounded in empirical investigation and considers the pedagogical and socio-economic contexts of higher education in LMICs is missing. Although Irugalbandara and Fernando’s (2019) work provides some perspective, its applicability appears limited, given it is designed to push vocational knowledge and the content delivered is at primary and junior secondary education levels.

Arguably, any of the models or frameworks from the context of educationally advanced countries may apply to learners in LMICs. However, caution should be taken in importing pedagogical solutions from educationally advanced to less-advanced countries due to differences in educational opportunities in the regions (Apiola & Tedre, 2012; Okai-Ugbaje, 2021). A good example is that many LMICs have a deficit in resources required to provide adequate pedagogical support to both faculty and students. Thus, the most practised and culturally familiar pedagogy is the didactic lecture, whereby students are mainly passive learners (Kuchah, 2018; Okai-Ugbaje, 2021). This stands in sharp contrast to the more student-centred pedagogies in advanced countries. Therefore, what works in educationally advanced countries may not be suitable in LMICs. This evidence suggests that an m-learning framework, in and from the context of higher education in LMICs, is necessary.

### Conceptual framework

The analysis in Table 1 provides rich insight into the pedagogical aspects of m-learning. However, the works of Koole (2009) and Ng and Nicholas (2013) stand out because of their approaches to theorising m-learning, explained below. Additionally, while each is unique, both frameworks have the strengths of other frameworks. On this basis, they provided a strong starting point for creating the conceptual framework. This study acknowledges the apparent contradiction of beginning with studies from the context of educationally advanced countries given the earlier argument. However, not giving them due consideration appeared short-sighted and negated the importance of building on existing knowledge.

**Fig. 1:**
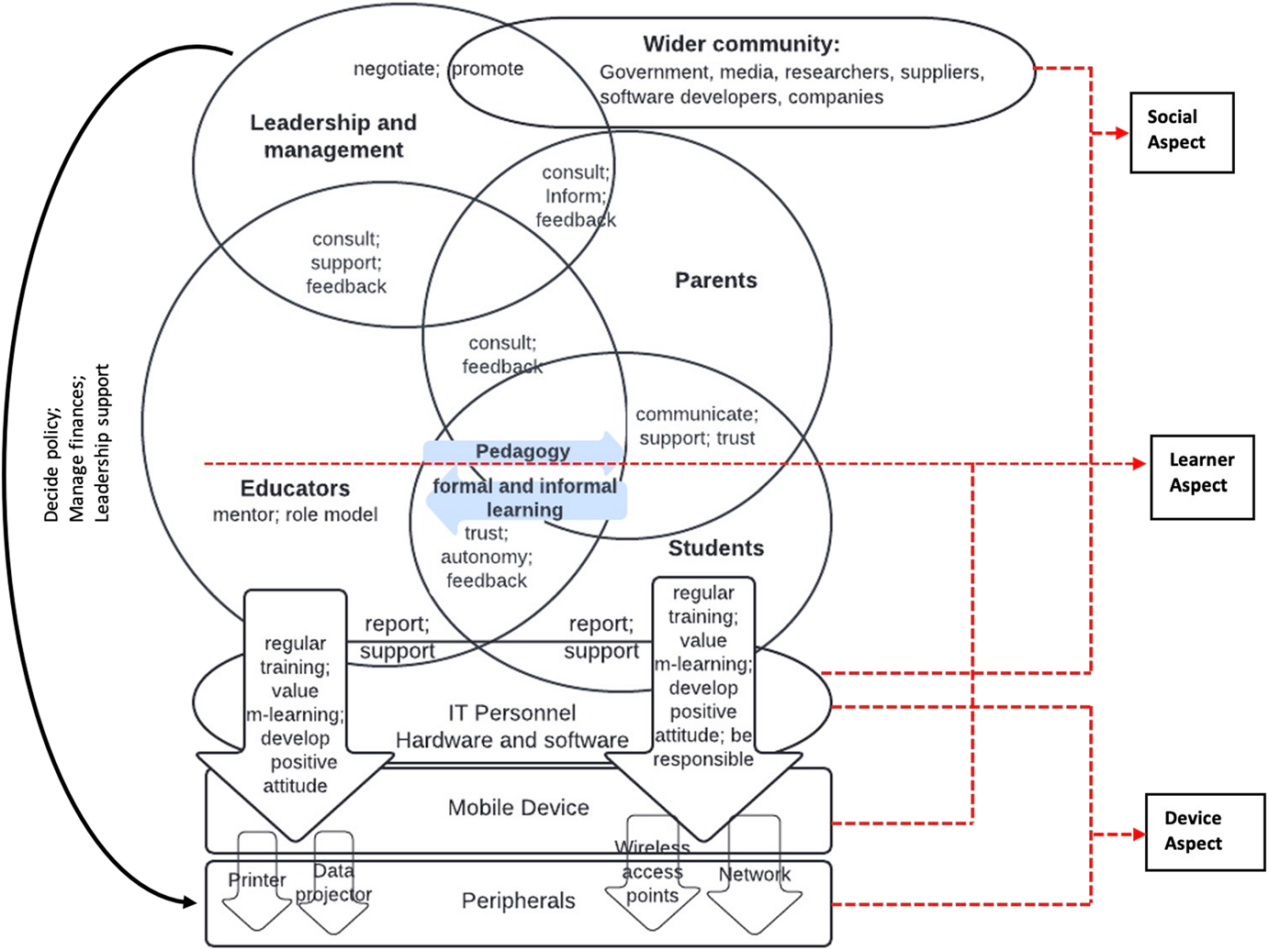
M-learning conceptual framework, informed by the combined works of Koole (2009) and Ng and Nicholas (2013)

Koole’s FRAME model (2009) considered the pedagogical, technocentric and interactive aspects of the mobile device for learning referred to as the learner, device, and social aspects respectively. A comparison of both theoretical approaches shows the core aspects of Koole’s framework are evident in Ng and Nicholas’s framework (2013). The learner aspect of the FRAME model considers the students’ prior knowledge and how that forms the basis for new knowledge, emphasising learning theories and how they affect the learner. Ng and Nicholas (2013) share this view, emphasising the pedagogical attributes of learning with handheld devices and the interpersonal relationship between students and educators; they posit that mobile devices not only can bridge formal and informal learning but also support seamless and long-term learning goals. The device aspect of the FRAME model represents the hardware and software characteristics of the mobile device and its usability for learning. While Ng and Nicholas (2013) share this foundation, they also emphasise other technological peripherals such as wireless access points, mobile networks and technical support from IT personnel as crucial for sustainable and seamless m-learning. Finally, the social aspect - the processes of social interaction and cooperation between stakeholders is essential for the sustainability of any m-learning initiative. These approaches towards theorising m-learning provide useful insight to this research, aimed at aligning theory with practice in ways that integrate the social, device and learner aspects of m-learning with the contextual realities of LMICs. Accordingly, Fig. 1, informed by both works, shows how the three aspects (social, learner and device) interconnect for a viable m-learning solution. It also highlights the importance of interaction between and among various stakeholder groups for successful m-learning.

## Creating the m-learning contextual framework for LMICs

Drawing inspiration from the conceptual framework presented in Fig. 1 and the takeaways from the analysed research outlined in Table 1, creating a contextual framework specific for LMICs is explored in the following section. Since context is critical, it begins with an overview of the pedagogical situation in many LMICs, then discusses how two learning theories provide the basis for a shift from current to potentially more effective pedagogical practices. The section concludes with a discussion of how exploiting local opportunities and mobile device attributes with effective stakeholder interaction could help manage some of the socioeconomic and sociotechnical challenges.

The most practised pedagogies in many higher institutions of learning in LMICs are the teacher-centred approach and traditional didactic face-to-face delivery, where educators are revered, and intellectual interchanges between students and educators are not widely practised (Apiola & Tedre, 2012; Damon et al., 2016; Muianga et al., 2018). Instead, students are passive recipients who memorise rather than conceptualise the content received (Okai-Ugbaje, 2021). In developing a framework for LMICs, it is essential not to ignore these realities but instead integrate technology to ensure synergy between existing practices and proposed solutions. As stated in the introduction, many higher learning institutions in LMICs have a limited basic educational infrastructure. This includes inadequately equipped computer laboratories and students’ limited ownership of personal computers (Akin, 2013; Damon et al., 2016; Eze et al., 2018). Acknowledging that m-learning cannot eradicate the need for these resources, the wide penetration of mobile technology and mobile device ownership by the vast majority of people in these countries (Silver, 2019), including higher education students, provide the possible avenue for m-leaning to be a viable alternative for web-based e-learning.

### Pedagogical considerations

To ensure synergy between existing practices and the successful integration of m-learning, it is beneficial to draw upon theories that view learning as a collaborative, engaging and motivating process. Arguably, these are essential attributes for meaningful learning regardless of the learning mode or delivery. While virtually all forms of learning can be made to foster collaboration and enhance students’ engagement, Laurillard (2007) argues that the intrinsic nature of mobile devices makes m-learning motivating because of the degree of ownership and control and opportunity to communicate with peers anytime, anywhere. This facilitates collaborative learning in ways otherwise difficult to achieve, and such collaboration could make learning fun. Further, for TEL to be both worthwhile and enjoyable, the learning design should be facilitated by principles and theories that ensure learning is situated, personal and encourages a high level of interaction with the learning context and content (Kearsley & Shneiderman, 1998). Two established theories that potentially provide these attributes, in addition to offering the appropriate level of challenge to stimulate meaningful learning and interaction, are the social constructivist theory (social constructivism) and the theory of optimal experience (Flow). Although both theories were developed decades before advances in educational technologies, they remain relevant as seminal theories used to effectively address the learning needs of twenty-first-century learners (Lockey et al., 2020; Singh et al., 2020). This is also evident in Table 1. Although a purposeful selection of studies, it shows that all studies underpinned by theory share a connection to seminal theories like constructivism. This is unsurprising given constructivism’s focus on student-centred learning - that is, a learning approach that actively engages students in the learning process through ‘student-student, student-content, student-instructor, and student-outside resources interactions’ and mobile devices’ potential as a learning tool to support the constructivist approach (Ozdamli, 2012, p. 929).

## Social constructivism

There is such a great overlap between constructivist and social constructivist theories (Jennings et al., 2013) that social constructivism is sometimes referred to as a subset of constructivism (Siemens, 2005). Regardless of their similarities, one distinct difference is social constructivist theory’s emphasis on interaction and collaboration in learning, and the view that knowledge is a human product that is first constructed in a social context, then internalised and used by the individual(s) (Amineh & Asl, 2015). The cardinal argument of social constructivism is that knowledge, as a product of human interaction, is mediated by tools or artefacts produced socially, culturally or technologically with which the learner can engage in learning (Baharom, 2013). Given the attributes of social constructivism, Cochrane and Bateman (2010) describe it as the strategic pedagogy underpinning higher education teaching and learning, arguing that applying its principles enables students to relate to what they learn and reflect their understanding by working in groups. That way, they can ‘refine their knowledge through arguments, structured controversy and reciprocal teaching and learning’ (Baharom, 2011, p. 6), leading to a shared understanding of the content.

## Theory of optimal experience (flow)

The inherent nature of the mobile device to keep users engaged warrants the inclusion of a theory that centres on intrinsic motivation to make learning enjoyable. The theory of optimal experience, called ‘Flow’, potentially offers such an outcome. Flow was coined by Csikszentmihalyi (1975), who defined it as ‘the holistic sensation that people feel when they act with total involvement’ (p. 36). The experience is often characterised by a deep concentration on and engagement in the activity with an intense sense of control, interest and enjoyment, which results in the individual losing track of time (Schmidt, 2010). The three conditions leading to Flow are: the activity has clear goals; there is a balance between challenge and skill; and immediate feedback is available (Csikszentmihalyi, 1990). Experiences characterised by such conditions have become known as the ‘Flow state’, denoting optimal experience in which the activity becomes worth doing for its own sake (Csikszentmihalyi, 1990). Flow in the educational context is associated with persistence in learning (Park et al., 2010), which can be influenced by intrinsic and extrinsic factors (Chang et al., 2018). Intrinsic factors include the learner’s personality and learning preferences, as well as the instructional design and how content is presented. Extrinsic factors include support and encouragement from peers and educators (Chang et al., 2018), which may be influenced through collaboration within and outside the learning environment. The application of Flow to m-learning could lead to active participation in the construction of knowledge and skill development (Power, 2013).

Applying the principles of social constructivism and Flow in designing m-learning opportunities has the potential to gradually bridge the cultural power distance between educators and students and provide students with a sense of control over their learning. According to Culbertson et al. (2015), another method for increasing student control is presenting material that leaves students feeling that learning is effortless. This is achievable when material is presented in a way that matches the students’ skill, it is viewed as understandable, and learning is likely to be effortless, leading to a sense of control to meet the demands of the course. Further, opportunities for immediate feedback may help maintain students’ focus and increase their interest in the learning activity (Park et al., 2010). Amineh and Asl (2015) and Ozdamli (2012) argue that these considerations provide the avenue for personalised, self-directed, lifelong learning. The integration of such an m-learning design as a blended approach to complement existing teaching and learning practices is one way to potentially introduce or strengthen technology-enhanced and student-centred learning in LMICs where such practices are either lacking or weak.

### Device attributes and stakeholder interaction

#### Mobile devices

In addition to pedagogical considerations, it is also important to consider how attributes of the mobile device impact m-learning design and integration. The connectivity and functionality of the mobile device and constant advancement in technology have transformed portable handheld devices (phones and tablets) from basic communication gadgets to service delivery platforms with tangible benefits and tremendous educational potential (Iqbal & Bhatti, 2020). Such advances make it possible to successfully expand educational opportunities and provide affordable solutions to educational problems, even to the world’s poorest nations, by leveraging the devices people already own (West & Vosloo, 2013). This is especially important in LMICs because m-learning initiatives in which participants are provided with devices are unlikely to be widespread due to the economic situations of low-income countries.

According to a 2019 Pew Research Centre report, mobile technology and smartphone ownership are increasing globally. However, while 83% of the surveyed population in emerging economies (nine countries) have mobile phones, only 45% own smartphones (Silver, 2019). Despite the relatively low level of smartphone ownership, most devices today have multimedia capabilities in addition to basic functionalities such as calling and texting. These device attributes provide the basis for m-learning. Moreover, studies have shown that the majority of higher education students in LMICs own phones with internet capability (Kaliisa & Picard, 2017; Lamptey & Boateng, 2017). Notably, some mobile phone manufacturers that target countries with low purchasing power are said to meet specific contextual needs in addition to being low-cost. For example, mobile devices (including smartphones) targeted at countries with unstable electricity supply are made to have longer battery life (Nsehe, 2017), with claims that some can run for up to five days before requiring a re-charge for an average phone user (Nkem-Gbemudu, 2016). These design features provide the avenue for m-learning to thrive. Moreover, using personal devices have been reported to increase students’ motivation to engage in m-learning and address their learning needs and desires, as they feel empowered ‘to make their own decisions facilitated by their own device’ (Bikanga Ada, 2018, p. 7), in addition to the ubiquitousness of mobile devices that is making learning and collaboration possible anytime, anywhere.

#### Stakeholders

As noted by Ng and Nicholas (2013), a key ingredient to the successful implementation of m-learning is involving stakeholders, such as leadership, management and IT personnel, in addition to students and educators. The role of these stakeholders, particularly management, is essential given effective leadership influences technology adoption by students and staff (Hauge & Norenes, 2015). Moreover, leadership that encourages productive relationships between educators and technical staff is vital, as the ability of the IT department and educators to successfully work together is crucial for achieving success in teaching and learning with technology (Salmon & Angood, 2013). The involvement of these stakeholders improves the quality of m-learning (Adedoja, 2016), and such alliances help educators develop the required skills for m-learning to work effectively (Handal, 2015). Further, Seong and Ho (2012) assert that even in the absence of advanced technologies, collective social capacity influenced by management has the potential to create an environment for communication, dialogue and collaboration among staff. This can foster sustainable policies and stimulate suitable approaches that take into account local capacity, culture and way of life suitable to the context. Accordingly, available resources used in a meaningful way have the potential for successful implementation. These considerations are especially important in LMICs where technological infrastructure is still evolving and to bridge the gap between policies and ideas that are too ambitious and those likely to succeed.

## Methods

### Overview

To evaluate and determine the practical application of the contextual framework, the authors conducted two separate investigations in a Nigerian university. Accounts of both studies are detailed in peer reviewed articles. The first, an exploratory study involved stakeholder groups in the conceptual framework (management, educators, students and IT personnel). The goal of the study was to determine the willingness and readiness of the stakeholders to use m-learning. Given the apparent difference in pedagogical practices between the existing teacher-centred approach at the university, and the technology-enhanced and student-centred learning attainable via m-learning, the study included a focus on the pedagogical readiness of educators and students to engage in m-learning. Interestingly, the findings showed that although all four stakeholder groups showed strong willingness and readiness for the implementation of m-learning, and students and educators were keen to engage in m-learning, some educators in spite of their support were concerned about losing control of the classroom if students became more independent learners. The empirical data and its analysis is reported in Okai-Ugbaje et al. (2020a). The authors concluded that the success of m-learning in practice would largely depend on the positive attitude of educators to the m-learning pedagogy.

Following the outcome of the exploratory study, an informal workshop was conducted in order to understand the concerns of educators regarding implementation including misconceptions about student-centred learning. The workshop helped concerned educators to see that enabling a democratic learning environment following the principles of social constructivism and Flow did not necessarily mean relinquishing control of the classroom, but rather provided students with the oppourtunity to be actively engaged participants in their learning journey. Following the workshop, two educators volunteered to trial m-learning, resulting in an experimental study of the practical implementation of m-learning (trial and intervention are used interchangeably when referring to the experimental study). The intervention had two goals. The first was to determine the impact of an m-learning design underpinned by the principles of social constructivism and Flow on the traditional face-to-face delivery and the learning experience of students, as well as the ability of educators to step back a little and let students take some control of their learning. The second goal was to determine the practicality of a cost-effective and sustainable m-learning delivery in an educational setting with limited educational technology resources. The intervention was undertaken using the blended learning approach in which m-learning was used as a complement rather than a standalone approach to augment the existing traditional delivery. Detailed accounts of how the studies were conducted, including how data were collected and analysed are reported in Okai-Ugbaje et al. (2020b).

### Research participants

Participants for both studies were students and staff from a Nigerian university. The exploratory study included 566 student participants and 21 staff members comprising 14 academics, four IT personnel and three senior management staff members. Participants for the second study included two academics and 208 students.

### Methodology

The methodology for both studies was a mixed-method design. It was chosen because a combination of both quantitative and qualitative data collection methods was considered most suitable to achieve the research goals. In the exploratory study, students’ responses were collected via survey and the other stakeholders’ views were collected via semi-structured interviews. In trailing the practical implementation of m-learning, the second study collected data via survey and observation of students. The m-learning component was delivered using lecture videos created by the course lecturers hosted over a public cloud platform. Students were required to watch the videos then collaborate on WhatsApp chat platforms created for the intervention, before the face-to-face class sessions. The WhatsApp platforms and the classroom served as data collection sites where the researcher was an observer. Further, students’ experiences from the intervention and thoughts about the trial were collected via survey after the study. The quantitative data from the exploratory and experimental studies were analysed using SPSS, and the qualitative data (semi-structured interviews) were analysed using the NVivo software.

## Reflections on the findings and discussion

**Fig. 2:**
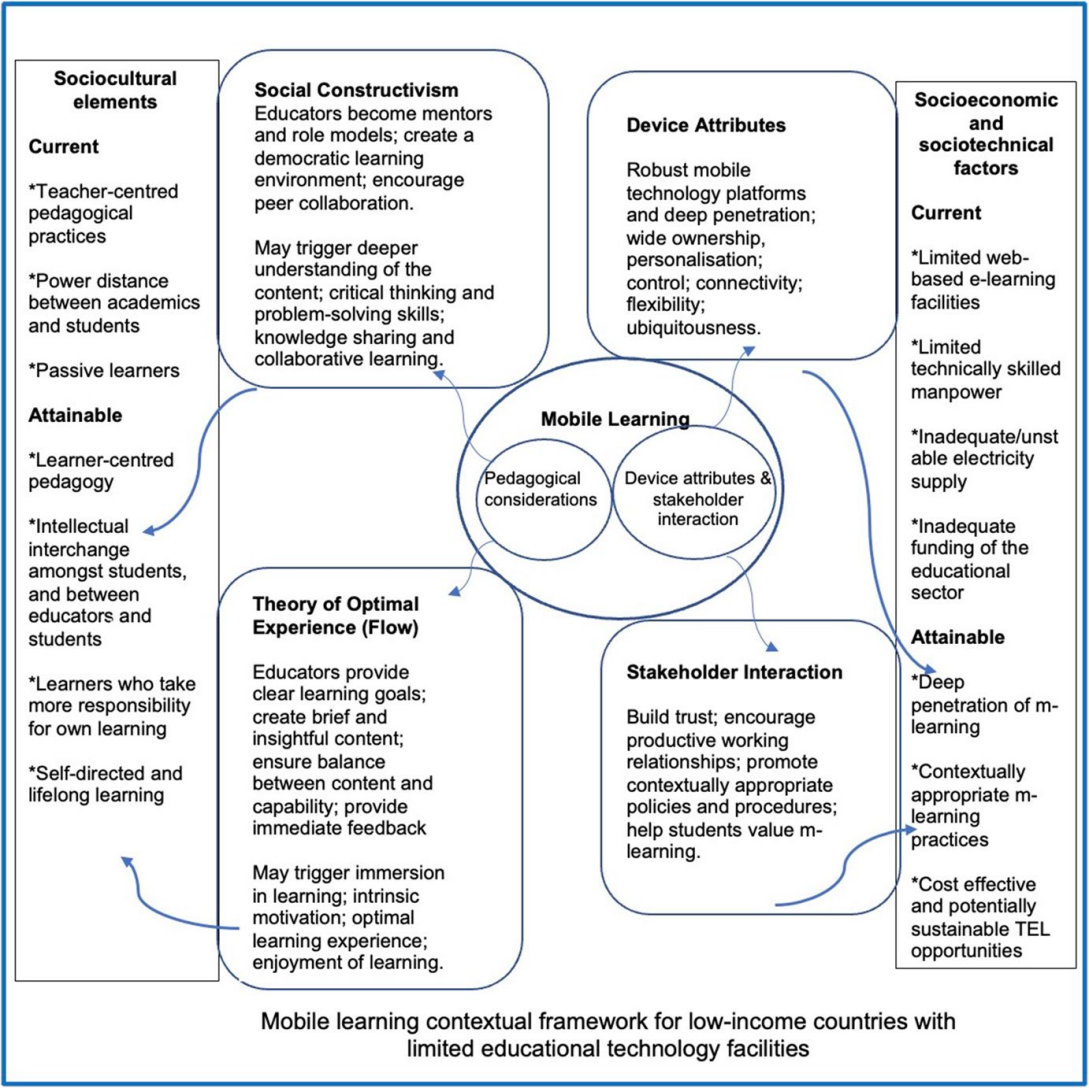
M-learning contextual framework for LMICs

This section reports on additional insights gained at the end of the project. Specifically, it shows how the contextual framework provides the pathway for a transition from current realities to what is attainable. Thus, this paper calls for m-learning research to go beyond trials and focus on mainstream integration, whereby m-learning is considered one of the main vehicles of higher education rather than an additional channel or enabler. This philosophical shift is particularly important in order to realise the unique potential of m-learning as a ‘catalyst for pedagogical change’ (Cochrane, 2014, p. 30). Building on the findings and experiences gathered from the exploratory and experimental studies, Fig. 2 provides a snapshot of current sociocultural elements, as well as socio-economic and sociotechnical factors that impact the integration of TEL in higher education in many LMICs. As noted earlier, this research does not claim that m-learning is the silver bullet to the challenges of TEL in LMICs. However, it does argue that a well-considered, contextually appropriate m-learning approach holds the potential to be a viable and effective alternative to web-based e-learning, where teacher-centred and traditional face-to-face approaches with limited technology use are the norms.

Similarly, the contextual framework (Fig. 2) also shows how applying the principles of social constructivism and Flow potentially provide the pathway for a shift from current pedagogical practices. Social constructivism advocates for dynamic interaction between educators and students and between the learning context and content, thereby promoting active learning. In other words, the learner can construct knowledge based on their active involvement in the learning process made possible through social interaction in a democratic learning environment enabled by the educator (Ozdamli, 2012). Thus, the teacher becomes a mentor or facilitator rather than delivering a didactic lecture where students are passive learners. Conversely, the principles of Flow advocate that educators provide clear learning goals and objectives so that students know expected learning outcomes (Chang et al., 2018; Power, 2013). In addition, the design parameters of Flow require learning to be at the appropriate skill level and content to be brief and concise to make learning engaging and fun. During the m-learning trial, the courses were presented in content grouped together via video (in approximately 15-minute chunks) and each lecture video was made to achieve a specific objective (Okai-Ugbaje et al., 2020b). This was especially important because of the relatively small screens of mobile devices. The combination of these principles to deliver some parts of the course provided the avenue for gradual and seamless integration of m-learning as a means of technology-enhanced and student-centred learning approach. Our study showed that the application of these principles saw students benefit from and value m-learning. For example, most students reported that content was easier to comprehend because the lecture videos made it possible for them to study at their own pace and convenience, with the opportunity to rewind as often as needed. Fostering a deeper understanding subsequently led students to ask more relevant and specific questions (Okai-Ugbaje et al., 2020b). In line with the findings of the trial, a growing body of evidence (Cho et al., 2015; Culbertson et al., 2015; dos Santos et al., 2018) shows that students who engage in collaborative learning environments underpinned by Flow exhibit a deeper understanding of the subject and better learning outcomes.

TEL integration goes beyond educators and students alone, and as a potentially sustainable mainstream m-learning approach, it requires institutional change management (Salmon & Angood, 2013). While a common success factor for IT interventions in developed countries is key stakeholders’ involvement in the project, the culture of many LMICs and power distance between the leaders and people require more than stakeholder involvement. The leadership’s conviction on the intervention and the importance of their role in influencing change is necessary for achieving desired results (Gregor et al., 2014). This parallels Pollack and Algeo’s (2015) argument that successful change management requires the organisation’s leadership and management to own and subsequently align the necessary change as required. Given the socio-economic and sociotechnical circumstances of many LMICs, including limited fixed broadband internet connectivity, which makes access to the internet predominantly through mobile broadband (International Telecommunication Union, 2017), m-learning may be facilitated through collaboration with local mobile network providers. The findings of the exploratory study suggest that collaboration with mobile service providers mediated through institutional management may boost alliances with the university’s IT department (IT personnel), including technical support to the university community and large data plans for students and staff at subsidised rates (Okai-Ugbaje et al., 2020a).

Considering LMCIs’ limited infrastructure capability, cloud-based m-learning was proposed and used for the trial. Cloud-based m-learning combines the benefits of cloud computing and m-learning, offering m-learning content delivery that is ubiquitous, convenient and low-cost, as it does not need any infrastructure purchases, installation, configuration and maintenance (Badidi, 2016; Masud & Huang, 2013; Simmon, 2018). It also eliminated obstacles, such as limited memory and processing power of mobile devices, often reported as limitations of traditional m-learning (Badidi, 2016; Masud & Huang, 2013). It further ensured continuity in learning even when students moved across multiple mobile devices. The outcome of the intervention strongly suggests that cloud-based m-learning, alongside device attributes such as personalisation and ubiquitousness, and strong stakeholder interaction that builds trust and promotes a mutual working relationship between administrative management, IT personnel and educators, could help to effectively manage the socio-economic and sociotechnical factors enumerated in Fig. 2. Further, as shown in the contextual framework, applying the principles of social constructivism and Flow as pedagogical underpinnings may help overcome current sociocultural factors that encourage teacher-centred pedagogical practices. For example, in adopting social constructivism, educators become mentors and role models rather than didactic teachers. In doing so, they create a democratic learning environment that encourages collaborative learning. These may trigger a deeper understanding of the content, leading to intellectual interchange between students and educators, and other learner-centred pedagogical practices. Likewise, in adopting Flow, educators provide clear learning goals and opportunities for feedback, which may trigger deeper engagement and enjoyment of learning.

## Conclusion, limitations and further research

The role of governments in educational systems is clearly important. However, current realities suggest that the governments of many LMICs who decades into the twenty-first century are still unable to provide basic needs like stable electricity, clean water, good sanitary conditions and other social amenities for the vast majority of their population; may not be able to provide robust educational systems as seen in educationally advanced climes anytime soon. Ironically, these limitations stand in sharp contrast to the deep penetration of mobile technology and wide ownership of mobile devices by the vast majority of the people in these countries, including students. This ironic situation can be capitalised upon, as it provides the avenue for m-leaning to be a viable alternative for web-based e-learning. Therefore, the opportunity arises for higher education policymakers and leaders to leverage mobile technology to design effective m-learning to facilitate TEL. In doing so, LMICs have the opportunity to leap-frog a generation of educational technologies used in the developed world and adopt m-learning directly, as it is more feasible.

We argue that the feasibility of m-learning holds great potential to improve learning conditions and expand the reach of higher education to a large population of LMICs currently deprived of such opportunities. This need has been accelerated by the realities of the COVID-19 pandemic when educational institutions are frantically looking for alternative means to traditional classrooms and face-to-face teaching. While m-learning has been widely acknowledged as a possible alternative, its implementation in many LMICs is still low despite the potential for success. Were m-learning a part of the educational system in many LMICs, the physical shutdown of institutions due to the pandemic may not have meant a halt in education, as was the case in many instances, until the resumption of face-to-face classes. Instead, a rapid and remote shift to m-learning may have been possible, as seen in countries where e-learning was an integral component of the educational system before the pandemic. Against this backdrop, this paper has presented a potential pathway for effective mainstream integration of m-learning in higher education in LMICs now and beyond the pandemic.

While this study has attempted to provide a contextual m-learning framework to guide sustainable m-learning in LMICs, it has some limitations. First, the study participants were drawn from only one university and included only internal stakeholders (students, educators, IT personnel and management). However, the study’s focus is a necessary first step in such an investigation, as it provides the basis for wider stakeholder participation. Future studies will do well to include external stakeholders, including mobile service providers. Second, this study only considered m-learning for teaching and supporting student learning. Further research on the role of m-learning in assessing and evaluating students’ performance is necessary for complete integration.

## Data Availability

The data that supports the findings of this study are available from the corresponding author upon reasonable request.

## Abbreviations

M-learning: Mobile learning
LMICs: Low- and middle-income countries.
TEL: Technology-enhanced learning.
COVID: Coronavirus disease.
